# Intrahepatic cholestasis of pregnancy can increase the risk of metabolic disorders: A meta-analysis

**DOI:** 10.5937/jomb0-33222

**Published:** 2022-10-15

**Authors:** Leiying Zhang, Chen Tang, Chenlian Ye, Luren Huang, Yan Wu

**Affiliations:** 1 The First Affiliated Hospital of Gannan Medical University, Department of Gynecology and Obstetrics, Ganzhou City, China

**Keywords:** intrahepatic cholestasis of pregnancy, preeclampsia, gestational diabetes mellitus, intrahepatčna holestaza trudnće, preeklampsija, gestacioni diabetes mellitus

## Abstract

**Background:**

Gestational diabetes mellitus (GDM) and preeclampsia (PE) are common complications during pregnancy. Studies indicated that abnormal bile acid metabolism is related to its pathogenesis. Intrahepatic cholestasis of pregnancy (ICP) is the most common pregnancy-specific liver disease, which classic symptoms include generalized pruritus that commonly and biochemical evidence of elevated bile acids. Our study aimed to explore the correlation between the ICP presence and risk of GDM, PE incident in pregnant women.

**Methods:**

A meta-analysis, which included 10 eligible studies including 17,688 ICP cases and 1,386,771 controls, was performed to assess the correlation of ICP with preeclampsia (PE) and gestational diabetes mellitus (GDM). There were 7 studies investigating the relationship between ICP and PE, and 9 studies that evaluated the relationship between ICP and GDM. All eligible studies were screened from Pubmed, Web of Science and EBSCO databases.

**Results:**

The results of this meta-analysis indicate that ICP significantly increase the risk for both PE (pooled odds ratio OR: 2.56 95%CI: 2.27 2.88, I2 heterogeneity = 35%, p heterogeneity = 0.16) and GDM (pooled OR: 2.28 95%CI: 1.69 3.07, I2 heterogeneity = 81%, p heterogeneity < 0.001). In the sensitivity analysis of GDM, excluding the largest heterogeneity study cannot change the result (pooled OR: 2.86 95%CI: 2.59 3.16, I2 heterogeneity = 0%, p heterogeneity = 0.56).

**Conclusions:**

This meta-analysis shows that ICP is closely associated with ICP increased risk of PE and GDM) during pregnancy.

## Abstract (sr)

**Uvod:**

Gestacijski dijabetes melitus (GDM) i preeklampsija (PE) su uobičajene komplikacije tokom trudnoće. Studije su pokazale da je abnormalni metabolizam žučne kiseline po vezan sa njegovom patogenezom. Intrahepatična holestaza u trudnći (ICP) je najčešća bolest jetre specifična za trudnoću, čiji klasični simptomi uključuju generalizovani svrab koji je uobičajen i biohemijski dokaz povišenih žučnih kiselina. Naša studija je imala za cilj da istraži korelaciju između prisustva ICP-a i rizika od GDM, PE incidenta kod trudnica.

**Metode:**

Urađena je meta-analiza, koja je uključivala 10 prihvatljivih studija uključujući 17,688 slučajeva ICP i 386,771 kontrola, da bi se procenila korelacija ICP-a sa preeklampsijom (PE) i gestacijskim dijabetesom melitusom (GDM). Bilo je 7 studija koje su ispitivale odnos između ICP i PE, i 9 studija koje su procenile odnos između ICP i GDM. Sve studije koje ispunjavaju uslove su pregledane iz baza podataka Pubmed, Web of Science i EBSCO.

## Introduction

Both preeclampsia (PE) and gestational diabetes mellitus (GDM) are common metabolic disorders during pregnancy, associated with neonatal and maternal mortality, increased hypertension and type 2 DM after pregnancy [1] [2]. Previous studies have reported that abnormal bile acid (BAs) metabolism at the early stage of pregnancy significantly contributes to metabolic disorders [3]. GDM is described as glucose intolerance that is first diagnosed in pregnancy. The pathogenesis has not been delineated. Studies in rodents and humans have shown an association between bile acids and Type 2 diabetes mellitus [4] [5], which has led to increased interest in understanding the role of bile acids in the development of GDM. Research has shown that farnesoid X receptor (FXR) expression is reduced in GDM, leading to abnormal glucose and bile acid metabolism [6]. FXR is a crucial gene that encodes proteins involved in bile acid synthesis; FXR is predominantly activated by primary bile acids, thereby regulating their synthesis and metabolism, Promoting the excretion and absorption of bile acids maintaining bile acid homeostasis. There is evidence that FXR is also involved in triglyceride and glucose metabolism [7]. Besides, it is plausible that reduced activity of FXR and Takeda G-protein receptor 5(TGR5) could be responsible for this increased susceptibility as evidence shows that both receptors are involved in glucose homeostasis [8] [9].

Intrahepatic cholestasis of pregnancy (ICP) is a pregnancy-specific condition identified by different degrees of pruritus, abnormal transaminases and elevated serum total BAs, affecting approximately 0.4~4% of pregnancies based on different regions [10] [11]. Even though ICP and PE and GDM are two distinct diseases, interestingly, they have similar abnormalities of BAs metabolism [4] [12]: 1. increased total cholic acid to chenodeoxycholic acid (CA/CDCA; i.e., FXR antagonists/agonists); 2. elevated primary conjugated Bas, particularly tauro-conjugated bile acids; 3. Potential functions of the TGR5 pathway. The current understanding of the hazards is limited to pregnant fetuses, but we do not eliminate maternal metabolic disorders. A recent study found that ICP is associated with impaired glucose tolerance and increased birth weight, which are features of GDM [13]. Besides, a study showed that women with ICP are at increased risk of developing GDM [14]. However, the opposite has been reported in other studies. Whether ICP can raise the risk of metabolic disorders in pregnancies remains unclear [15] [16]. This meta-analysis aims to provide evidence of evidence-based medicine that ICP can increase the risk of metabolic disorders in pregnancies. Moreover, through this analysis, we will also provide new insight into the potential role of abnormal BAs metabolism, even in non-metabolic liver diseases, in promoting metabolic disorders.

## Materials and methods

### Search strategy

Reviewed studies were screened from PubMed, Web of Science and EBSCO database published from 1 January 2000 to 1 January 2018, with the following medical subject headings (MeSH): intrahepatic cholestasis of pregnancy, obstetric cholestasis; preeclampsia, eclampsia, pregnancy-induced hypertension, gestational hypertension; gestational diabetes mellitus, gestational diabetes; clinical trial, case-control and cohort study [14] [17] [18] [19] [20] [21] [22] [23] [24] [25] [26]. The search only included articles written in English. We also perform manual searches to avoid missing eligible studies. When other articles reported the results of one article simultaneously, only the most original article was included.

### Inclusion and exclusion criteria

The included studies followed the following standards: 1) the clinical manifestation (i.e., pruritus without any other causes) and the laboratory examination (i.e., elevated serum total BAs) are necessary for the case/exposure definition; 2) absence of ICP is the primary condition for the control or no-exposure definition; 3) clear definition of the outcomes: PE and GDM; 4) clear and separate data for the outcomes: PE and GDM. Studies were excluded if they lacked a clear definition for the case/exposure, control/no-exposure, outcomes, and clear and independent data of outcomes [27] [28] [29] [30] [31] [32].

### Assessment of quality

The Newcastle-Ottawa Quality Assessment Scale was used to evaluate the quality of included studies. Two researchers participated in the evaluation of studies quality. Scores [1] [2] [3] [4] [5] [6] [7] [8] were generalized for the fitness for inclusion into this meta-analysis, according to population selection, the comparability of the outcomes and the definition of the exposure/no-exposure factor, according to population selection, the comparability of the outcomes and the definition of the exposure/no-exposure factor. Studies with a score < 5 were considered to be of low quality [33] [34] [35] [36] [37] [38] [39].

### Data extraction and statistical analysis

Data were extracted from the number of GDM or PE in ICP and no-ICP to represent the outcome of ICP. In addition, the first author's name, publication year, country, and the numbers of cases/exposure and controls/no-exposure, as defined in inclusion and exclusion criteria, were also included. The metaanalysis was performed by Revman 5.3.3 (Cochrane Collaboration, The Nordic Cochrane Centre, Copenhagen) and STATA 16.0 (College Station, TX, USA). Association of PE or GDM with ICP was estimated using crude odds ratios (OR) with 95% confidence intervals (95% CI), and P < 0.05 was considered to be statistical significance. All crude ORs were pooled through a random-effects model for generating conservative conclusions. Heterogeneity test was performed by a Chi-square-based Q test and I^2^ statistic. P < 0.05 and I^2^ > 50% indicate significant heterogeneity. Sensitivity analyses were used to assess metaanalysis results' stability by removing included studies one by one. Begg's and Egger's tests were used for quantificational evaluation of publication bias. Funnel plots were used with In ORs and selnORs to visualize publication bias. Revman 5.3.3 (Cochrane Collaboration, The Nordic Cochrane Centre, Copen hagen) was used to calculate the pooled OR and evaluate the sensitivity, and STATA 16.0 (College Station, TX, USA) was used to assess publication bias.

## Results

### Characteristics of included research

**Figure 1:**
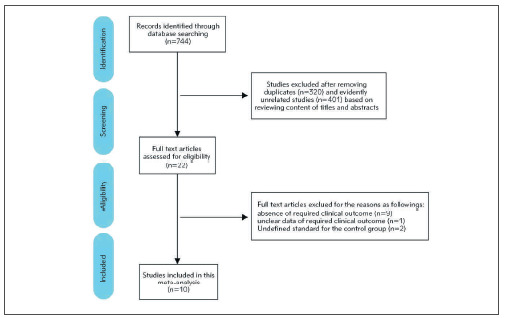
Flow chart of the study selection procedure. A total of nine studies were included in our study.

A total of 10 articles [36] [37] [38] [39] [40] [41] [42] [43] [44] [45] [14] from 744 records in Pubmed, Web of Science and EBSCO database were included in this meta-analysis. The strategy of literature filtering and selection is summarized in Figure 1. The basic features and quality evaluation of these selected pieces of literature are presented in Table 1-Table 2. Briefly, this meta-analysis includes 1,404,459 subjects, of whom 17,688 with the diagnosis of ICP are served as the case or exposure group, while 1,386,771 without ICP are used as the control or non-exposure group, from 9 retrospective cohort studies and 1 retrospective case-control study. The time of these studies is from 2008 to 2016, which cover 8 countries: Canada, United States, Sweden, Turkey, Israel, India, United Kingdom and China. The research population among three of 10 studies is not representative, in which the targeted population all have multiple pregnancies or liver diseases of pregnancy, and the targeted population in the Chinese cohort is not stratified according to the critical factor: assisted reproductive technology (ART). Thus, the assessment scores in these three studies are lower than the others.

**Table 1:** Characteristics of included Studies.

Study	Country	Study<br>type	Sample<br>size (n)	ICP<br>No. (n)	No-ICP<br>No. (n)	Maternal age<br>(years, x̄ ± SDor IQR)	Gestational ageat delivery<br>(weeks, x̄ ± SD)	Complicated<br>GDM(n)	Complicated<br>PE(n)
Lausman 2008 [38]	Canada	Cohort	263	11	252	33.8 ± 5.1 inno-ICP; 36.5± 4.9 in ICP	34.1 ± 4.2in no-ICP; 34.7± 1.8 in ICP	NA	17 in no-ICP;1 in ICP
Allen 2015 [39]	UnitedStates	Cohort	247	26	221	NA in no-ICP;29 (26~31)in ICP	NA in no-ICP;37.5 (37~38)n ICP	19 in no-ICP;4 in ICP	NA
Shemer 2013 [40]	Sweden	Cohort	1,213,668	5477	1,208,191	NA	NA	11,468 inno-ICP; 146 inICP	33,539 inno-ICP; 364in ICP
Shemer 2015 [41]	Sweden	Cohort	125,281	11,388	113,893	29.01 ± 5.34in no-ICP; 29.01± 5.34 in ICP	NA	901 inno-ICP; 267 inICP	3302 inno-ICP; 854in ICP
Yerebasmaz 2016 [42]	Turkey	Cohort	260	56	204	27.5 ± 5.8 inno-ICP; 28.7 ±5.1 in ICP	38.9 ± 1.5in no-ICP;38.1±1.2in ICP	13 in no-ICP;9 in ICP	9 in no-ICP;7 in ICP
Erkenekli 2015 [43]	Turkey	Cohort	412	103	309	NA	NA	16 in no-ICP;12 in ICP	9 in no-ICP;4 in ICP
Raz 2015 [44]	Israel	Cohort	378	78	300	31.67 ± 5.11in Singletonno-ICP; 33.42± 5.48 inSingletonICP33.41 ±4.75 in Twinsno-ICP; 34.41± 5.27 in Twinsno-ICP	39.49 ± 1.54in Singletonno-ICP; 37.16± 1.3 inSingletonICP36.3±2.69in Twinsno-ICP; 34.84± 2.25 inTwins no-ICP	15 inno-ICP; 11 inICP	9 inno-ICP; 12 inICP
Arbinder 2010 [45]	India	Cohort	5347	47	5300	NA	NA	111 inno-ICP; 3in ICP	NA
Martineau 2014 [14]	UnitedKingdom	Case control	57,131	140	56,991	NA	NA	4861 inno-ICP; 19in ICP	NA
Shan 2016 [37]	China	Cohort	1472	362	1110	30.23 ± 4.8 inno-ICP; 30.80± 4.73 in ICP	NA	279 in no-ICP;104 in ICP	88 in no-ICP;51 in ICP

**Table 2:** Quality assessment of included studies.

Cohort Study									
Study ID	Selection				Comparability	Exposure			Scores
	Representativeness of the expose cohort	Selection of the nonexposed cohort	Ascertainmentof exposure	Not any outcomeat start of study	Study controls for important factors	Assessment of outcome	Enough follow-up time for the occurrence of outcomes	Adequacyof follow-up of cohorts	
Lausman 2008 [38]	0	1	1	1	1	1	1	1	7
Allen 2015 [39]	0	1	1	1	1	1	1	1	7
Shemer 2013 [41]	1	1	1	1	1	1	1	1	8
Shemer 2015 [41]	1	1	1	1	1	1	1	1	8
Yerebasmaz 2016 [42]	1	1	1	1	1	1	1	1	8
Erkenekli 2015 [43]	1	1	1	1	1	1	1	1	8
Raz 2015[44]	1	1	1	1	1	1	1	1	8
Arbinder 2010 [45]	1	1	1	1	1	1	1	1	8
Shan 2016[37]	0	1	1	1	0	1	1	1	6
Case Control									
Study ID	Selection				Comparability	Exposure			Scores
	Adequate casedefinition	Representativeness of the cases	Selection of controls	Definition of controls	Study controls for important factors	Ascertainment of exposure	Same method of ascertainment for	Non-response rate	
Martineau 2014 [14]	1	1	1	1	1	1	1	1	8

### ICP and PE

**Figure 2:**
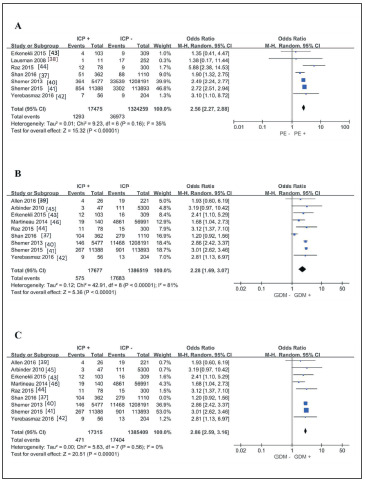
Forest plots using a random-effects model: A) the relationship between ICP and PE, B) the relationship between ICP and GDM, and C) the relationship between ICP and GDM by excluding the most extensive heterogeneity study.

The data of PE merged into ICP, and no-ICP groups are presented in 7 studies [37] [38] [40] [41] [42] [43] [44]. Pooled Odds ratios from these 7 studies reveal that ICP significantly increases the risk of PE (pooled OR: 2.56 (95%CI: 2.27~2.88), I^2^_heterogeneity_ = 35%, p _heterogeneity_ = 0.16) (Figure 2A). Each time, sensitivity analysis by removing included articles reveals an unchanged result, indicating that this result is reliable.

### ICP and GDM

There are 9 studies [37] [39] [40] [41] [42] [43] [44] [45] [14] that listed the number of ICP and no-ICP complicated with GDM. The pooled analysis results of these 9 studies are shown in Figure 2B. Obviously, ICP also increases the risk of GDM (pooled OR: 2.28 (95%CI: 1.69~3.07)) but with high heterogeneity (I^2^_heterogeneity_ = 81%, p _heterogeneity_ < 0.001). Although sensitivity analysis cannot reverse this conclusion, we still focused on the source of heterogeneity. By ruling out Shan's study, we observed that heterogeneity is completely eliminated, and the conclusion (pooled OR: 2.86 (95%CI: 2.59~3.16), I^2^_heterogeneity_ = 0%, p _heterogeneity_ = 0.56) (Figure 2C) is consistent with the original conclusion. By reviewing this article that causes significant heterogeneity, we noticed that ART was used in both ICP and no-ICP, and the rate of ART (ICP vs. no-ICP: 191/362 vs. 486/1110, p < 0.05) had a significant difference between the two groups. Moreover, ART in their study was considered as an independent risk factor for GDM (OR: 1.66, 95%CI: 1.32~2.10). Considering the influence of this confounding factor (ART) on the result, we believe it is inappropriate to include this study for calculating pooled OR of GDM.

### Publication bias

**Figure 3:**
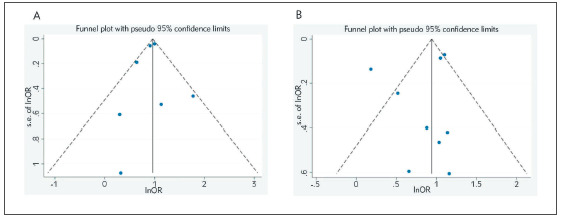
Funnel plots using a random-effects model: A) publication bias of the relationship between ICP and PE and B) publication bias of the relationship between ICP and GDM.

Funnel plots of two results from this meta-analysis are symmetrical (Figure 3A-Figure 3B). Begg's and Egger's tests indicate that there is no obvious publication bias in the analysis of association of ICP with PE (Begg's test: z = 0.00, p = 1.000; Egger's test: t = -0.49, p = 0.646) or GDM (Begg's test: z = 0.31, p = 0.754; Egger's test: t = -0.74, p = 0.484).

## Discussion

In this meta-analysis, which included 10 eligible studies including 17,688 ICP cases and 1,386,771 controls, we found that ICP can increase the risk of metabolic disorders (i.e., PE and GDM) during pregnancy.

ICP is transient and generally follows a benign course in gravidas but may adversely affect the prognosis of the fetus. The primary adverse fetal outcomes that have been reported include PTB, MSAF, fetal distress, RDS, and asphyxia [26]. The most dreadful complication gravidas with ICP will experience is intrauterine fetal death without early warning signs. The mechanism underlying the pathogenesis of ICP and the mechanisms by which ICP leads to poor fetal outcomes are unclear. Recent study findings demonstrated significantly increased risks of adverse perinatal outcomes in gravidas with severe ICP [27]. Besides, the study included 7 kinds of research, 1293 out of 17475 ICP pregnant women had PE, using a random-effects model show some correlation between ICP and PE. Preeclampsia is a severe complication of pregnancy where it affects 5-8% of all pregnancies. Although the pathogenesis of PE has not yet been fully elucidated, endothelial dysfunction is considered to be the core event in its pathogenesis. Endothelial dysfunction disrupts normal vasoconstriction and diastolic function and increases vascular permeability, thus leading to hypertension and proteinuria [19]. Recent studies have confirmed that Sphingosine-1-phosphate receptor 2(S1PR2) played an essential role in maintaining the endothelial cell function, especially in vascular permeability and tension [20] [21]. Activating the S1PR2 signal disrupts the vascular function by activating Rho-associated kinase (ROCK) signaling and causing the imbalance in contraction and diastolic vascular media [22] [23].

S1PRs, including S1PR1-5, are a family of G protein-coupled receptors with sphingosine 1 phosphate (S1P) as their primary ligand [24]. A more recent study has shown that the natural ligands of S1PR2 not only include S1P, while conjugated BAs can also function a variety of biological effects by specific combination with S1PR2 rather than other S1PRs [25]. Although there is a lack of direct evidence that conjugated BAs can mediate the development of PE through S1PR2, the potential link between elevated S1PR2 agonists (i.e., conjugated BAs) and activating S1R2 signals in the pathogenesis of PE provides a possible explanation for the increased risk of PE among ICP patients, which own a significantly elevated serum conjugated BAs spectrum [26].

The pathogenesis and pathophysiology of GDM include increased estrogen, progesterone, hormones, adipokines and cortisol during pregnancy. Insulin resistance plays a vital role in the development of GDM [27] [28]. The study included 9 types of research, 575 out of 17677 ICP pregnant women had GDM, using a random-effects model show some correlation between ICP and GDM. The first study confirms that ICP is related to GDM in pregnant women. It has been well studied that BAs mediated FXR signaling is involved in insulin resistance. Lack of FXR signal leads to abnormal glucose and lipid metabolism and insulin resistance, involved in the pathogenesis of type 2 DM [38] [39]. Moreover, exogenous FXR agonists administration can significantly improve insulin resistance in human beings [7]. There is no doubt that the pathogenesis of GDM is still centered on insulin resistance. Further, the level of fibroblast growth factor 19 (FGF19), as a downstream molecule of intestinal FXR signal, is significantly decreased in GDM and exhibits a negative correlation with insulin resistance [31]. Since the components of BAs exhibit the different potential on FXR activation: free and conjugated CDCA > DCA = LCA > CA [40] [41] [42] [43], free and conjugated CA/CDCA is usually used to reflect the state of FXR activation and inhibition in the previous study [44] [45] [14]. Because of BAs spectrum in ICP, which is not only characterized by elevated conjugated BAs (S1PR2 agonists), but also increased total CA/CDCA (FXR antagonists), and the role of FXR signaling in GDM, it is not difficult to understand the contribution role of ICP in GDM development.

In the present study, we found that the primary source of heterogeneity was derived from the Shan et al. study [37], especially regarding the description of ART. This study showed that ART was used in both ICP and no-ICP, and the rate of ART had a significant difference between the two groups. Moreover, ART in their study was considered an independent risk factor for GDM. Considering the influence of this confounding factor on the result, we reject the study.

### Limitation

Since ICP is a late pregnancy disease, often diagnosed at the 30th week of gestation, we do not know the sequence of metabolic disorders and ICP in included studies. So the increased risk in this metaanalysis refers to the risk of prevalence rather than the incident risk.

## Conclusion

In summary, we find that ICP also increases the risk of metabolic disorders (i.e., PE and GDM) through this meta-analysis. This study provides evidence-based medical evidence for the increasing risk of PE and GDM in ICP, provides new insight into the role of abnormal bile acid metabolism in promoting metabolic disorders, and provides a new idea for the prevention of gestational diabetes mellitus and preeclampsia clinicians.

## Dodatak

### List of Abbreviations

GDM, Gestational diabetes mellitus;<br>PE, Preeclampsia;<br>ICP, Intrahepatic cholestasis of pregnancy;<br>Bas, bile acid;<br>FXR, Farnesoid X receptor;<br>TGR5, Takeda G-proteinreceptor 5(TGR5);<br>OR, odds ratio;<br>S1PR2, Sphingosine-1-phosphate receptor 2.

### Acknowledgments

The authors thank those who collected data or performed the measurement.

### Funding

This research did not receive any specific grant or funding from any commercial or nonprofit organization or public agency.

### Ethics statement

Formal institutional review board approval was not required as this manuscript only addresses data extracted from already published studies.

### Conflict of interest statement

All the authors declare that they have no conflict of interest in this work.
